# Evaluation of quality of life and recurrence rate after intraperitoneal onlay mesh laparoscopic repair of ventral hernia with or without closure of the defect (pIPOM vs. sIPOM)

**DOI:** 10.3389/fsurg.2026.1907864

**Published:** 2026-08-25

**Authors:** F. Pepe, G. Bottero, M. Giaccone, R. Bellino, E. Gambali, G. Balestra, S. Rosati, S. Sandrucci

**Affiliations:** 1 Università Degli Studi di Torino, Turin, Italy; 2 Azienda Ospedaliero Universitaria (AOU) Città della Salute e della Scienza, Presidio Ospedaliero Molinette, Chirurgia dei Sarcomi e dei Tumori Rari Viscerali, Turin, Italy; 3 ASL NO, Novara, Italy; 4 Politecnico di Torino, Turin, Italy

**Keywords:** IPOM plus, laparoscopic, quality of life, recurrence, ventral hernia

## Abstract

**Purpose:**

We want to evaluate the influence of defect closure in either primary or secondary laparoscopic ventral hernia repair in terms of both recurrence rate and peri- or postoperative hernia-related QoL.

**Methods:**

In this retrospective, non-randomized single-center trial, we analyzed 124 patients who underwent a laparoscopic repair for either a primitive or incisional ventral hernia with sIPOM(Group 1) or pIPOM(Group2) between February 2018 and May 2024. All patients were subjected to a specific questionnaire for the assessment of postoperative QoL in hernia-related patients (Eura-HS QoL) prior to surgery and at 6 months after the hernia repair.

**Results:**

The two groups did not show significant differences in key baseline and hernia-related characteristics. The median time of follow-up was 49.5 months for the sIPOM group, and 30.5 months for pIPOM Group (*P* value < 0.001). Recurrence occurs only in the sIPOM group (11.9% vs. 0%, *p* value=0.001). Postoperative QoL scores show an important improvement in every patient. While both techniques demonstrated similar post-operative pain levels, pIPOM had a significantly higher rate of aesthetic satisfaction. Subgroup analysis within the pIPOM group did not show significant differences based on the addition of diastasis repair.

**Conclusion:**

In conclusion we believe the closure of the defect to be an effective and safe method to improve the clinical and functional outcomes in laparoscopic repair of ventral hernia.

## Introduction

Ventral hernia, either primary or secondary (incisional), is a very common condition affecting many people worldwide. The exact prevalence of these conditions is still unknown, but it was estimated to be 0.5%–10% for the primary ventral hernia and 10%–12% for incisional hernia in pathology studies (1, 2).

Several surgical approaches have been described for the repair of the ventral hernia. Nowadays, laparoscopic intraperitoneal onlay mesh (IPOM) and sublay mesh repair either. With retromuscolar or preperitoneale mesh placement with an open anterior approach or with a minimally invasive technique (like eTEP or PeTEP) are common techniques for the treatment of primary and secondary abdominal wall hernias. Many studies were made over the years comparing the more traditional open techniques to laparoscopic ones without showing an absolute advantage of either one in both short and long-term outcomes (3–5). Currently, both sublay repair or intraperitoneal hernia repair could be considered safe and feasible treatments with similar recurrence rates for primary or secondary ventral hernia repair. The latest guidelines suggest laparoscopic ventral and incisional hernia repair (LVIHR) to be preferable for obese patients with defects between 3 and 10 cm in diameter, given the better outcomes in terms of wound infection and seroma formation (6–11). On the contrary, LVIHR seems to be burdened with a higher intraoperative complication rate and more technical difficulty (12). Long-term quality of life (QoL) does not differ between laparoscopic and open incisional or ventral hernia repair, but on the other hand there is a Level 1B evidence that Short-term QoL is better after laparoscopic repair compared to open anterior repair (9). Since its first description by LeBlanc in 1993, the standard IPOM (sIPOM) technique has evolved over the years (13). Recently more authors proposed laparoscopic closure of the defect prior to mesh positioning, denominated as IPOM plus (pIPOM). The pIPOM has been introduced in the guidelines for the laparoscopic treatment of ventral and incisional abdominal wall hernias published by the International Endohernia Society (IEHS) (7, 9, 10) as an effective way to reduce the recurrence rate, seroma formation and mesh bulging, despite being more time-consuming and technically challenging (14–20). The latest guidelines for treatment of Midline incisional hernia of the European Hernia Society also state that fascial closure during LVIHR is associated to lower recurrence rate and lower risk of hematoma and seroma formation (21). A concern about the pIPOM technique is the theoretically higher risk of postoperative and chronic pain that could affect the short-term and long-term postoperative QoL.

Above all the literature only few authors inspect this argument. Clapp et al. assessed the chronic pain, the overall and cosmetic satisfaction in a 10-point scale and the functional outcomes with a small Activity Assessment Scale (AAS) questionnaire. The study didn't show any difference in terms of chronic pain, but it shows better outcome in term of satisfaction and activity assessment in favour of pIPOM (22). Saijo et al. administered a generic QoL questionnaire to 33 patients who underwent a laparoscopic repair with or without defect closure (23). The study showed that the laparoscopic technique improved QoL, regardless of defect closure. Recently, a prospective multicenter trial was published from an Italian group, showing no significant differences in post-operative Gastrointestinal Quality of Life Index (GIQLI) between sIPOM and pIPOM technique (24). However, the QoL after sIPOM and pIPOM remains unclear, and no current analyses using a hernia-related and validated QoL Questionnaire have been performed.

## Materials and methods

This is a retrospective, non-randomized single-center trial. We analyzed 124 patients who underwent a laparoscopic repair for either a primitive or incisional ventral hernia with standard IPOM (sIPOM) or IPOM plus (pIPOM) between February 2018 and May 2024. Excluding criteria for the study were: patients under 18 years of age, pregnancy, poor preoperative health status (ASA ≥ 4 - a scoring system of the American Society of Anesthesiologists), radio or corticosteroid therapy at the time of surgery, and emergency cases.

All data were gathered through clinical records, phone interviews, and clinical examinations. We collected anamnestic clinical and instrumental data for each patient: age, sex, BMI, previous surgeries, physical status according to the ASA score, and hernia characteristics in type (primary or secondary), location, and the number of defects, mesh type and size. Hernia characteristics were grouped according to the European Hernia Society (EHS) classification as W1 (< 4 cm), W2 (≥ 4–10 cm) or W3 (≥ 10 cm) and hernia location was grouped as medial [subxiphoidal (M1), epigastric (M2), umbilical (M3), Infraumbilical (M4), suprapubic (M5)], lateral [subcostal (L1), flank (L2), iliac (L3), lumbar (L4)], or combined (25). Diastasis Recti was defined as a width between the two medial edges of the abdmonis recti greater than 3 cm.

Intraoperative or postoperative complications, and recurrence, were collected, as well as the total length of hospitalization. Recurrence was defined as the clinical or instrumental (either with ultrasound tomography or computed tomography scans) finding of a hernia in the same place as the previous repair, regardless of the need for surgical repair.

**Figure 1:**
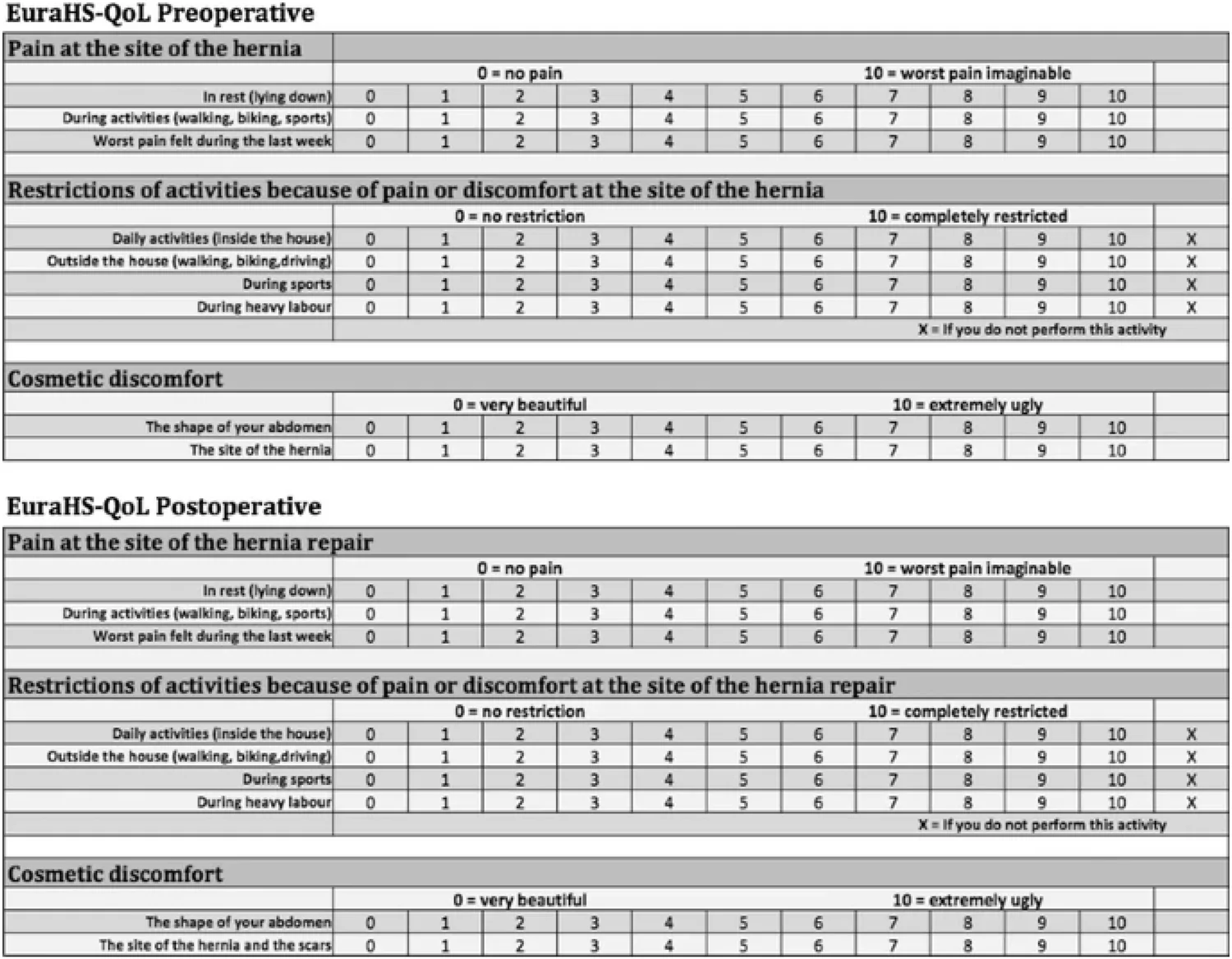
EuraHS quality of life questionnaire paper administered to patients.

Complications were reported according to the Clavien-Dindo classification (26). All the patients were asked to undergo a validated hernia-specific QoL questionnaire: the EuraHS-QoL (27). QoL questionnaire was filled prior to surgery and 6 month after the hernia repair and evaluated pain, restrictions in daily activity, and aesthetic discomfort. The total score in each time period ranged from 0 (best QoL) to 90 (worst QoL). All domains and questions of the EuraHS-QoL can be found in Figure 1. Patients were divided into the sIPOM group (Group I) and pIPOM group (Group II). The trial's main endpoint was the recurrence rate and differences in QoL between groups. Secondary endpoints were complications and hospital stays.

A subgroup analysis was conducted within the pIPOM group, including patients who also underwent a diastasis recti repair and those who did not. The aim was to better assess the impact of transparietal stitches on post-operative QoL.

### Surgery

All procedures were performed by an experienced surgeon who had performed at least 50 laparoscopic repairs. The laparoscopic repair was performed as follows. Pneumoperitoneum was induced through a Verres needle in the upper left quadrant. After Palmer's test was performed, a 12 mm trocar was then inserted in the left lateral region at the intersection between the umbilical line and the midaxillary line. Two other operative 5 mm trocars were inserted on the same line, a few centimeters below the left costal margin and above the left iliac spine. Adhesiolysis and hernia content reduction were performed when needed using either an electrocautery hook or bipolar, until the hernial defect was correctly exposed and measured. Partial peritonectomy was then performed above and below the hernia defect with the section of the round ligament and of the urachus when needed, to promote tissue integration and better adherence of the mesh and avoid mesh sliding. When pIPOM was performed, the hernia defect was closed with some transparietal stitches of #0 or #1 absorbable suture (Polyglactin). Diastasis recti repair was obtain using similar transparietal stitches at the rate of one every centimeter. A double-layer non-absorbable, light surgical polypropylene prosthesis, composed of a macroporous monofilament mesh and a transparent adhesion-free film mesh (2P-PCMC, DIPROMED s.r.l., San Mauro Torinese (TO), Italia) with at least 5 cm overlap of the defect was then selected. Before inserting the mesh into the abdomen, the mesh was stitched with 4 threads at the 4 cardinal extremities and then rolled and inserted through the 12-mm trocar. The mesh was then centered on the defect and a Reverdin needle was used for the transparietal fixation of the four cardinal points. Further fixation was achieved with absorbable tackers (Sorbafix, BARD, RI, USA). After the trocars' extraction, the 12-mm fascial incision was closed. In the end, compressive medication was performed.

### Statistical analysis

Continuous variables were reported as mean and standard deviation (SD) for normally distributed variables and as the median and interquartile range (IQR) for non-normal distributions. Categorical variables were referred to as frequencies and percentages. Shapiro- Wilk normality tests were used to assess normality distribution.

Univariate analysis of the difference between groups was performed with the nonparametric Mann–Whitney test for continuous variables and with the chi-squared test (with Fisher correction when needed) for categorical data. For all the used tests the statistical significance level was set at the conventional *p* < 0.05.

The results were analyzed using the Statistics and Machine Learning Toolbox of MATLAB® release 2024a (The Math-Works Inc., Natick, MA, USA).

### Ethical board

This trial was approved institutional board of AOU Città della Salute e della Scienza based in Turin (Italy) on the date of 24/04/2025. Consent was obtained from each patient in the study.

## Results

A total of 124 patients were included in the study. Of these, 42 were in the sIPOM group and 82 in the pIPOM group.

Baseline patient and hernia characteristics, including sex, BMI, ASA score, number of defects, hernia type, dimension, and location, were comparable between the two groups, with no statistically significant differences. However, the pIPOM group was significantly older than the sIPOM group (median age 59 [33; 81] vs. 65 [36; 92], *p* = 0.032). All these results are summarized in Table 1.

**Table 1:** Comparison of charateristics between sIPOM and pIPOM groups.

Characteristic	sIPOM (*n* = 42)	pIPOM (*n* = 82)	*p*-value
Gender			0.915
Male	25 (59.5%)	48 (58.5%)	
Female	17 (40.5%)	34 (41.5%)	
Age (median, range)	59 (33; 81)	65 (36; 92)	**0** **.** **032**
BMI (mean ± SD)	30.05 ± 5.48	28.17 ± 4.32	0.088
Obesity			0.130
Yes	18 (42.9%)	24 (29.3%)	
No	24 (57.1%)	58 (70.7%)	
ASA Score			0.555
I	9 (21.4%)	15 (18.3%)	
II	28 (66.7%)	51 (62.2%)	
III	5 (11.9%)	16 (19.5%)	
IV	0 (0%)	0 (0%)	
Hernia Type			0.192
Primitive	21 (50%)	31 (37.8%)	
Incisional	21 (50%)	51 (62.2%)	
Defect Number			0.274
1	32 (76.2%)	71 (86.6%)	
2	7 (16.7%)	9 (11.0%)	
≥ 3	3 (7.1%)	2 (2.4%)	
Hernia position			0.267
M1-M2	15 (35.7%)	23 (28%)	
M3	16 (38.1%)	30 (36.6%)	
M4-M5	1 (2.3%)	6 (7.3%)	
L	4 (9.5%)	17 (20.7%)	
Multiple	6 (14,2%)	6 (7.3%)	
Hernia Dimension			0.782
W1	18 (42.8%)	30 (36.6%)	
W2	22 (52,4%)	47 (57.3%)	
W3	2 (4,77%)	5 (6.1%)	
Mesh overlap (median, range)	11 (8; 26)	10 (7–16)	0.083
Follow-up (median, range)	49 (5; 66)	30.5 (3; 65)	**<0**.**001**
Hospital Stay (median, range)	2 (1; 5)	2 (1; 6)	0.67
Recurrence			**0**.**001**
Yes	5 (11.9%)	0 (0%)	
No	37 (88.1%)	82 (100%)	
Post-operative complications			0.67
No	39 (92.8%)	76 (92.7%)	
Yes	3 (7.2%)	6 (7.3%)	
Complications Clavien-Dindo			0.293
0	39	76	
1	0 (0%)	4 (5.3%)	
2	1 (2.5%)	1 (1.3%)	
3	2 (5.12)	1 (1.3%)	

The median follow-up time was longer in the sIPOM group than in the pIPOM (49 months vs. 30.5 months, *p*-value <0.001). No difference were found in the median hospital stay between the two groups at 2 days (*p*-value=0.67). Mesh overlap was calculated for each patient and was always superior to 5 cm and similar between the groups.

Post-operative complication rates at 9.5% in the sIPOM group and 7.3% in the pIPOM group (*p*-value=0.67).

The recurrence rate was 11.9% (5 patients) in the sIPOM group, while no recurrence was observed in the pIPOM group (*p*-value=0.001).

The EuraHS-QoL questionnaire was used to assess quality of life (QoL) before and after the procedures. Both the total score and the specific scores of every domain (pain, restriction in activities, and aesthetics) were evaluated. Both surgical techniques led to a significant improvement in the overall QoL of patients after the LVIHR. An inter-group analysis revealed no statistical difference in pre-operative pain and total QoL scores. However, the pIPOM group showed significantly better pre-operative scores for the “Restriction in Activities” domain (20 [0–29] vs. 23 [0–40], *p*-value=0.023). Post-operatively, there were no significant differences between the groups regarding pain or restriction in activities. A significant difference was found in aesthetic results, with the pIPOM group demonstrating a better aesthetic outcome compared to the sIPOM group (0 [0–17] vs. 0 [0–15], *p*-value < 0.001). This difference, despite similar median scores and ranges, was attributed to a significantly higher proportion of patients in the pIPOM group reporting an optimal aesthetic result (85.36%), scored as zero, compared to the sIPOM group (52.38%). Ultimately, when considering the total post-operative scores, no significant difference between the two surgical techniques was observed. All these data are summarized in Table 2.

**Table 2:** Quality of life comparison between sIPOM and pIPOM groups.

QoL Domain (median, range)	sIPOM (*n* = 42)	pIPOM (*n* = 82)	*p*-value
Pre-operative Pain	21.5 (0; 28)	21 (0; 28)	0.511
Post-operative Pain	2 (0; 15)	3 (0; 17)	0.802
Pre-operative Aesthetics	7.5 (0; 15)	6 (0; 18)	0.436
Post-operative Aesthetics	0 (0; 17)	0 (0; 15)	**<0** **.** **001**
Pre-operative Restriction in Activities	20 (0; 29)	23 (0; 40)	**0**.**022**
Post-operative Restriction in Activities	1.5 (0; 17)	4 (0; 13)	0.731
Pre-operative Total QoL	47.5 (4; 71)	48.5 (9; 80)	0.317
Post-operative Total QoL	5.5 (0; 44)	6 (0; 44)	0.86

Furthermore, a subgroup analysis was conducted within the pIPOM group to better investigate whether the addition of a major suture line (diastasis recti repair) could influence short- and long-term outcomes. Subgroup analysis can be found in Table 3. Patients who underwent a diastasis repairs had a higher median BMI (29 vs. 27.5, *p* value = 0.02) and a higher percentage of primitive hernia (56.5 vs. 30.5, *p* value 0.03). No difference was found regarding median length of hospital stay, post-operative complications and recurrence rate. As shown in Table 4 no significant differences were found between the subgroups (diastasis repair vs. no diastasis repair) regarding postoperative outcomes or QoL scores.

**Table 3:** Comparison of charateristics between groups of patients in whom diastasis repair was or was not performed.

Characteristic	No Diastasis Repair (*n* = 59)	Diastasis Repair (*n* = 23)	*p*-value
Gender			0.205
Male	32 (54.2%)	16 (69.6%)	
Female	27 (45.8%)	7 (30.4%)	
Age (median, range)	66.5 (37;92)	61 (36;81)	0.375
BMI (mean ± SD)	27.5 (18; 46.5)	29 (25.1; 35.2)	**0** **.** **02**
ASA Score			0.55
I	12 (20.3%)	3 (13%)	
II	37 (62.7%)	14 (60.9%)	
III	10 (17%)	6 (26.1%)	
Hernia Type			**0**.**03**
Primitive	18 (30.5%)	13 (56.5%)	
Incisional	41 (69.5%)	10 (43.5%)	
Defect Number			0.381
1	53 (89.8%)	18 (78.2%)	
2	5 (8.5%)	4 (17.4%)	
≥ 3	1 (1.7%)	1 (4.3%)	
Hernia position			**0**.**02**
M1-M2	12 (20.3%)	11 (47.8%)	
M3	20 (33.9%)	10 (43.5%)	
M4-M5	6 (10.2%)	0 (0%)	
L	16 (27.1%)	1 (4.3%)	
Multiple	5 (10.2%)	1 (4.3%)	
Hernia Dimension			0.375
W1	24 (40.6%)	6 (26.1%)	
W2	31 (52.5%)	16 (69.6%)	
W3	4 (6.8%)	1 (4.3%)	
Mesh overlap (median, range)	10 (7;16)	11 (8;15)	0.250
Hospital Stay	2 (1; 6)	3 (1; 5)	0.086
Recurrence			-
Yes	0	0	
No	59	23	
Follow-up	14 (5; 65)	34 (3; 59)	**0**.**044**
Post-operative complications			0.52
No	54 (91.5%)	22 (95.6%)	
Yes	5 (8.5%)	1 (4.4%)	
Complications Clavien-Dindo			0.25
0	54 (91.5%)	22 (95.6%)	
1	4 (6.7%)	0	
2	1 (1.7%)	0	
3	0	1 (4.4%)	

**Table 4:** Quality of life comparison between groups of patients in whom diastasis repair was or was not performed.

QoL Domain (median, range)	No Diastasis Repair (*n* = 59)	Diastasis Repair (*n* = 23)	*p*-value
Pre-operative Pain	20 (0; 28)	21 (7; 28)	0.403
Post-operative Pain	3 (0; 17)	3 (0; 15)	0.611
Pre-operative Aesthetics	8 (0; 18)	4 (0; 11)	0.10
Post-operative Aesthetics	0 (0; 15)	0 (0; 5)	0.69
Pre-operative Restriction in Activities	23 (0; 40)	21 (10; 32)	0.70
Post-operative Restriction in Activities	4 (0; 12)	2 (0; 13)	0.64
Pre-operative Total QoL	49 (9; 80)	48 (25; 69)	0.57
Post-operative Total QoL	6 (0; 44)	6 (0;29)	0.58

## Discussion

The issue of fascial defect closure in laparoscopic repair has long been debated. Traditionally, the sIPOM technique has been associated with a higher incidence of recurrence, seroma formation, and postoperative bulging (14, 15, 17, 19, 20, 28).

The aim of closing of the defect is to restore the integrity of the abdominal wall before the mesh positioning, allowing a greater mesh overlap without leaving the intraperitoneal mesh bridging the defect. For those reasons also the International Endohernia Society (IEHS) and European Hernia Society (EHS) guidelines also recommend the fascial closure in the LVIHR, even if the strength of the recommendation is weak (9, 21). Our findings are consistent with previous studies. We found a significant difference in the recurrence rate of 11.9% in the sIPOM Group over a zero recurrence rate in the pIPOM Group (*p* value = 0.001), despite no difference was found in the overlap of the mesh in the groups. This difference seems highly relevant, even if we must consider that the higher follow-up time in the sIPOM group could be a potential source of bias, reducing the robustness of our finding.

According to a previous study (21), pIPOM seems to have a lower rate of complications, especially regarding seroma formation. Those findings are not confirmed by our study, where no significant difference was assessed in overall post-operative complications between groups. Seroma formation wasn't collected separately, so we cannot make definitive statement regarding that topic.

The ideal method of fascial closure technique is not yet standardized. The IEHS guidelines recommended the use of non-absorbable sutures, but no statement regarding which type of techniques has ever been made (7). Defect closure could be done either intracorporeally with a running suture or extracorporeally, of which different techniques are described in the literature (29–31).

The main concern of the pIPOM is that the increasing tension on the abdominal wall could lead to a higher post-operative pain, especially when the closure stitches are given transparietally as we do in our practice. On the other hand, there is the theoretical concern that bridging technique of sIPOM without fascial and abdominis recti muscle approximation could worsen the postoperative outcome. In fact, Den Hartog et al. (32) showed a significantly lower isokinetic strength of the trunk flexor after hernia repair with sIPOM when compared to open technique with fascial approximation and defect closure. The authors hypothesized that midline incisional hernias displace the rectus muscles laterally that could lead to a weakened abdominal muscle strength and to higher postoperative back pain. The first attempt to assess QoL difference between sIPOM e pIPOM was done by Clapp at al. (22). The authors in fact recorded the post-operative chronic pain rate at 6 months and both overall and cosmetic satisfaction assessed using a 10-point, Likert-type scale (1 = least satisﬁed, 10 = most satisﬁed). They also assessed the overall functional status with a series of 13 questions in accordance with the Activities Assessment Scale (AAS). The study showed no differences in terms of postoperative pain but significant better results in the pIPOM group in terms of overall (8.8 ± 0.4 vs. 7.0 ± 0.5; *p* = 0.008) and cosmetic (8.8 ± 0.4 vs. 7.0 ± 0.6; *p* = 0.01) satisfaction and in postoperative functional score (79.1 ± 1.9 vs. 71.3 ± 2.3, *p* = 0.002).

On the other hand, in a recent prospective trial conducted by Pizza et al. (24) no difference in terms of QoL was found. The authors recorded the functional results using the Gastrointestinal Quality of Life Index (GIQLI) a 36-item questionnaire that addresses five domains: upper gastrointestinal symptoms, lower gastrointestinal symptoms, physical status, psychological status and social status. Cosmesis satisfaction was assessed in a phone interview at 36 months in a yes or no answer fashion. The GIQLI index showed a great improvement after the surgery in both groups without any significant difference, but it didn't include specific pain domain. Regarding the cosmetic effect, pIPOM and sIPOM showed similar results (92.8% vs. 88.7%, *p* = 0.330).

By our knowledge the best attempt to consider the impact of the sIPOM and pIPOM on the post-operative quality of life was done by Saijo at al. (23). Their main concern was about the postoperative pain, and they utilized a validated QoL questionnaire including a pain domain, the SF36. Their results showed that quality of life improved in all the patients who underwent LIVHR, either sIPOM or pIPOM. Furthermore, sIPOM and pIPOM seemed to not differ in any of the considered QoL outcomes. Especially postoperative chronic pain recorded one year after surgery was the same in both groups. However, they used a very comprehensive but non-specific questionnaire for ventral hernia assessment, and a main limitation of the study lied in the small sample size of only 33 patients.

While the QoL after LVIHR has been a subject of previous research, the methodologies employed have varied. Prior investigations have frequently adapted general-purpose questionnaires, which may not fully capture the nuanced experiences of patients undergoing these specific procedures. Furthermore, the lack of assessment of key domains in the used questionnaire like postoperative pain, activity restriction, and cosmetic outcomes has often lacked the granularity required to better evaluate hernia-related factor and post-operative outcome. By using the EuraHS-QoL specifically validated for this patient population, we wanted to overcome these limitations and reduce the possible underestimation of differences between the sIPOM and pIPOM techniques.

In line with previous studies our results show a clear improvement in quality of life for patients undergoing either the sIPOM or pIPOM procedure.

The analysis of the total pre- and post-operative scores, as well as that of the single domains concerning pain, the restriction of various activities at rest or under effort and aesthetics, shows that all the patients undergoing LIVHR have an improvement in the hernia specific QoL.

The comparison of post-operative outcomes between the two groups by taking into consideration the single questionnaire domains shows that the two techniques do not differ either as regards post-operative pain or as regards overall restriction. The same results could be seen even comparing people who underwent a recti diastasis repair, that usually required a higher number of transparietal stitches. This result, like all the previous studies assessing chronic pain, seems to reassure that the closure of the defect, even with a considerable number of stitches and greater traction, does not increase the risk of chronic pain.

In line with the study of Clapp et al. but in contrast with that of Pizza et al., we found a difference in post-operative aesthetics results in favor of the pIPOM. Our findings seem to show only a slight advantage of pIPOM in terms of aesthetic satisfaction in patients. However, a careful examination of the data reveals a significant difference in the rate of maximum post-operative aesthetic satisfaction. In fact, there is a much greater number of patients in the pIPOM group who report a value of “zero” which corresponds to the evaluation “extremely beautiful” in both questions regarding the 'Shape of abdomen' and the 'state of hernia' in the post-operative questionnaire (85.4% vs. 52.3%).

Therefore, our data show how the apposition of stitches to close the defect or to repair the abdominal diastasis does not significantly affect the postoperative quality of life but seems to guarantee better clinical outcomes by significantly reducing the recurrence rate.

## Limitation

Our study has several inherent limitations that warrant consideration. The primary limitation is its retrospective and non-randomized design, which inherently carries the risk of selection and confounding biases. A second significant limitation is the discrepancy in follow-up duration between the two cohorts, being significantly longer in the sIPOM group (49 months) compared to the pIPOM group (30.5 months). Given that recurrences tend to manifest over time, this temporal difference could have skewed our recurrence rate analysis; although no recurrences were observed in the pIPOM group during the study period, an extended follow-up might yield different outcomes. Furthermore, we did not stratify postoperative Quality of Life (QoL) outcomes according to hernia defect size. Performing this stratification could have provided deeper clinical insights into the functional recovery of patients, particularly those presenting with larger (EHS W3) defects. Finally, the sample size—specifically the relatively small number of patients who underwent sIPOM—represents an additional limitation. Nevertheless, it should be emphasized that our overall cohort remains one of the largest to date investigating this specific topic.

## Conclusion

We believe the closure of the defect to be an effective and safe method to improve the clinical and functional outcomes of LVIHR. However, given the retrospective nature of our study and the bias caused by the different follow-up times between the two groups, a randomized controlled trial with a larger sample size is warranted to further validate our findings and overcome the limitations of this study.

## Data Availability

The raw data supporting the conclusions of this article will be made available by the authors, without undue reservation.
